# A single gene controls leaf background color in caladium (Araceae) and is tightly linked to genes for leaf main vein color, spotting and rugosity

**DOI:** 10.1038/hortres.2016.67

**Published:** 2017-01-04

**Authors:** Zhe Cao, Shunzhao Sui, Qian Yang, Zhanao Deng

**Affiliations:** 1 Gulf Coast Research and Education Center, Department of Environmental Horticulture, IFAS, University of Florida, 14625 County Road 672, Wimauma, FL 33598, USA; 2 College of Horticulture and Landscape, Southwest University, Chongqing 400715, China; 3 Visiting Scientist, Gulf Coast Research and Education Center, Department of Environmental Horticulture, IFAS, University of Florida, 14625 County Road 672, Wimauma, FL 33598, USA

## Abstract

Modern cultivated caladiums (*Caladium*×*hortulanum*) are grown for their long-lasting and colorful leaves. Understanding the mode of inheritance for caladium leaf characteristics is critical for plant breeders to select appropriate parents, predict progeny performance, estimate breeding population sizes needed, and increase breeding efficiencies. This study was conducted to determine the mode of inheritance of two leaf background colors (lemon and green) in caladium and to understand their relationships with four other important leaf characteristics including leaf shape, main vein color, spotting, and rugosity. Seven caladium cultivars and three breeding lines were used as parents in 19 crosses, and their progeny were phenotyped for segregation of leaf traits. Results showed that the two leaf background colors are controlled by a single nuclear locus, with two alleles, *LEM* and *lem*, which control the dominant lemon and the recessive green leaf background color, respectively. The lemon-colored cultivar ‘Miss Muffet’ and breeding lines UF-52 and UF-53 have a heterozygous genotype *LEMlem*. Chi-square tests showed that the leaf background color locus *LEM* is independent from the leaf shape locus *F*, but is tightly linked to three loci (*S*, *V* and *RLF*) controlling leaf spotting, main vein color, and rugosity in caladium. A linkage map that consists of four loci controlling major caladium leaf characteristics and extends ~15 cM was developed based on the observed recombination frequencies. This is the first report on the mode of inheritance of leaf background colors in caladium and in the Araceae family. The information gained in this study will be very useful for caladium breeding and study of the inheritance of leaf colors in other ornamental aroids, an important group of ornamental plants in the world.

## Introduction

Caladiums are often grown in containers, hanging baskets, and landscapes for their long-lasting and colorful leaves. Modern commercially grown caladium cultivars seem to have originated from two species, *Caladium bicolor* and *Caladium schomburgkii*.^1^ The primary method that has been used to develop caladium cultivars is sexual hybridization between elite cultivars and breeding lines.^2^ Large-scale commercial production of caladium plants has been mainly through tuber division. Currently, approximate 95% of caladium tubers used in the world are produced in Florida.

The most important factor determining the ornamental value of commercial caladiums is their leaf characteristics. Understanding the mode of inheritance of important leaf characteristics is very critical to caladium breeding and genetic improvement. Such knowledge can enable breeders to select appropriate parents for breeding, estimate population sizes required to achieve particular breeding goals, predict the likely segregation patterns of traits in progeny, and improve breeding and selection efficiency. Recent efforts toward the study of inheritance of caladium leaf characteristics have resulted in a number of important findings. It has been reported that leaf shapes in caladium are controlled by a single locus with two co-dominant alleles *F* and *f*, resulting in three genotypes (*FF*, *Ff* and *ff*) that control the fancy, lance, and strap-shaped leaves, respectively.^3^ Caladium main vein colors are regulated by a single nuclear locus *V* with three alleles [*V*^r^ (for red veins)>*V*^w^ (for white veins)>*V*^g^ (for green veins)].^3^ Leaf spotting in caladium is controlled by a single nuclear locus with a dominant allele *S* for spotting and a recessive allele *s* for non-spotting.^4^ Similarly, leaf blotching is also regulated by a single nuclear locus with two alleles; *B* for blotched leaves is completely dominant to *b* for non-blotched leaves.^5^ A recent study has shown that leaf rugosity in caladium is controlled by a single nuclear locus with a dominant allele *RLF* for rugose leaves and a recessive allele *rlf* for non-rugose or flat leaves.^6^

Leaf background color is another important leaf characteristic in caladium. The leaf background color in caladiums can be broadly grouped into two categories, green and lemon. Lemon-colored caladium leaves are light yellow throughout the growing season regardless of the light levels (from full sun to partial shade) and temperatures under which caladium plants are grown. The great majority of commercial caladium cultivars have a green leaf background color, although shades of green may exist.^7,8^ The lemon background color, in combination with bright burgundy, red, or pink spots or blotches, has resulted in a number of attractive, highly valued caladium cultivars. Therefore, there has been a consistent interest in incorporating the lemon background color into new cultivars.

Information on the mode of inheritance for the leaf background color in caladium has been lacking; so has been in the whole Araceae family. Different modes of inheritance have been reported for leaf background colors in some other foliage plants. Roberts *et al.*^9^ reported that the leaf background color in Eastern redbud (*Cercis canadensis*) was controlled by a single locus with a dominant allele for the purple background color and a recessive allele for the green background color. The yellow and green leaf background colors in *Sambucus nigra* are controlled by a dominant and a recessive allele, respectively.^10^ The green leaf background in barberries (*Berberis* spp.) was controlled by a recessive allele while the red leaf background was regulated by a dominant allele.^11^ Reportedly, two loci control the leaf background color in hazelnut trees.^12^

The objectives of this study were to (1) understand the mode of inheritance for the leaf background colors in caladium, (2) infer the leaf background color genotype of important caladium cultivars and (3) determine the genetic relationships of leaf background colors with four other important leaf characteristics including leaf shape, main vein color, spotting, and rugosity.

## Materials and methods

### Plant materials

**Figure 1:**
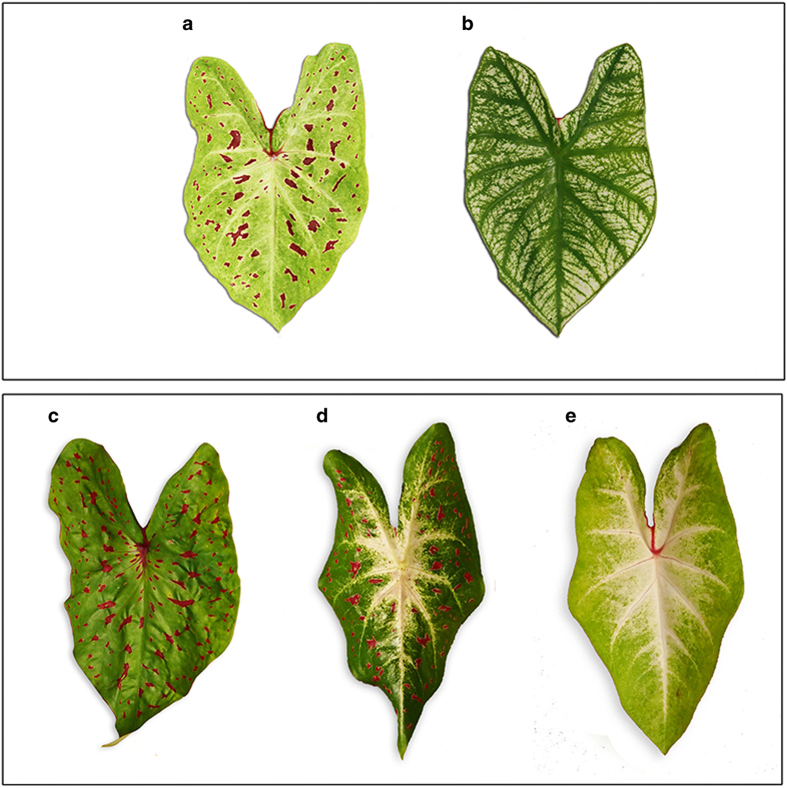
Typical leaves of ‘Miss Muffet’ (**a**: fancy, rugose, spotted, white-veined, and lemon background color), ‘Candidum’ (**b**: fancy, non-rugose, non-spotted, green-veined, and green background color), and three progeny [(**c**: fancy, rugose, spotted, green-veined, and green background color), (**d**: fancy, non-rugose, spotted, white-veined, and green background color), and (**e**: fancy, non-rugose, non-spotted, white-veined, and lemon background color)] from the crosses between ‘Miss Muffet’ and ‘Gingerland’.

Seven cultivars and three breeding lines were used as parents in 19 crosses (Table 1). Commercial cultivar ‘Miss Muffet’ and breeding lines UF-52 and UF-53 exhibited a lemon background color (Figure 1); commercial cultivars ‘Aaron’, ‘Candidum’, ‘Fairytale Princess’, ‘Gingerland’, ‘Fla. Moonlight’, and ‘Red Flash’ and breeding line UF-317 are characterized by a green leaf background color. These cultivars and breeding lines were often used in caladium breeding programs to develop new caladium cultivars with enhanced aesthetical values, improved plant performance and biotic and abiotic tolerance. Their phenotypes and inferred genotypes for leaf spotting, shape, main vein color, rugosity and leaf background color were shown in Table 1.

### Flower induction and controlled crossing

Induction of caladium flowers was conducted by immersing jumbo-sized tubers (6.4–8.9 cm in diameter) in a 600 mg·L^−1^ gibberellic acid (GA_3_) solution (ProGibb T&O; Valent BioSciences, Libertyville, IL, USA) at room temperature for 16 h in May 2012.^13^ Four to six GA_3_-treated tubers per parent were grown in plastic pots (20 cm in diameter) filled with a soilless potting mix (Fafard 3B; Conrad Fafard, Agawam, MA, USA) amended with 5 g of controlled-release fertilizer (Osmocote, 18N-2.6P-10K; The Scotts Company, Marysville, OH, USA). All caladium parental plants were grown on metal benches in a greenhouse with the temperature between 23 and 30 °C at the University of Florida’s Gulf Coast Research and Education Center (UF/GCREC), Wimauma, FL, USA. From early Jul. to mid-Sept. 2012, fresh pollen was collected from staminate flowers and stored in a refrigerator at 4 °C.^14^ Controlled pollinations were conducted 1 or 2 days before anthesis (unfurling of the spathe). Pollinated flowers were bagged until the fruit was mature.

### Seed germination and progeny growing

Seeds were manually extracted from mature berries. Dried seeds were immediately sowed in 20-row germination trays filled by a commercial soilless substrate (Fafard Super Fine Germination Mix) and germinated in a growth chamber with a constant temperature of 25 °C and continuous light (cool fluorescent lights, 30 μmol·m^−2^·s^−1^). After one month, seedlings were individually transplanted to 128-cell trays filled with a commercial substrate (Fafard 3B) and grown in a greenhouse. Young plants were irrigated by hand once a day and fertilized twice a week using a commercial water-soluble fertilizer containing 1.1% (w/w) ammonia nitrogen, 11.8% (w/w) nitrate nitrogen, 2.1% (w/w) urea nitrogen, 5% (w/w) phosphate (P_2_O_5_), and 15% potassium (K_2_O) (Peters Excel; Everris NA, Dublin, OH, USA). Between Apr. and May 2013, plants were transferred to raising beds at the GCREC experimental farm under a seepage irrigation system. Each plant was fed with 7.5 g of controlled-release fertilizer (Osmocote, 18N-2.6P-10K).

### Phenotyping

While grown in the field, caladium progeny were phenotyped for two leaf background colors (lemon or green), two leaf shapes (fancy or lance), leaf spotting (present or absent), three main vein colors (red, white or green), and leaf rugosity (present or absent) between Jun. and Sept. 2014. The phenotype of each progeny was examined at least four times and confirmed by at least two persons.

### Data analysis

Segregation of leaf background color as well as leaf shape, main vein color, spotting, and rugosity in various caladium populations was examined by chi-square test for goodness of fit against expected Mendelian segregation ratios. Contingency chi-square tests were conducted for identification of possible independence or linkage between loci using the program developed by Preacher.^15^ To calculate the recombination frequency between traits, the number of recombinant progeny was divided by the total number of progeny in a population and multiplied by 100. Recombination frequencies were then converted to genetic distances in centiMorgan (cM) using the Kosambi’s mapping function {*m*=¼ ln [(1+2*r*)/(1−2*r*)]×100}, where m represents genetic distance between loci and *r* is recombination frequency.

## Results

### Inheritance of leaf background color in caladiums

When ‘Miss Muffet’ was selfed (Table 2; cross no. 1), progeny segregated in a ratio of 3 (lemon): 1 (green) (*P*=0.521). This segregation ratio suggests that the leaf background color is controlled by a single nuclear locus with a dominant allele for the lemon background color and a recessive allele for the green background color, and that ‘Miss Muffet’ should be heterozygous at this locus. When ‘Miss Muffet’ was crossed with ‘Gingerland’ or ‘Candidum’ (Table 2; cross no. 2–5), their progeny segregated in a ratio of 1 (lemon): 1 (green) (*P*=0.265–0.685). This segregation ratio was observed in crosses ‘Miss Muffet’×‘Red Flash’, ‘Aaron’×‘Miss Muffet’, ‘Fla. Moonlight’×‘Miss Muffet’ (Table 2; cross no. 5–7) (*P*=0.167–0.577). These results suggested that maternal factors were not involved in the inheritance of leaf background color.

When green-colored parents, including ‘Gingerland’, ‘Candidum’, ‘Red Flash’, ‘Fla. Moonlight’, ‘Fairytale Princess’, ‘Aaron’ and UF-317, were crossed (Table 2; cross no. 9–16), their progeny all exhibited green leaves, suggesting that these parents are homozygous recessive for leaf background color.

Two breeding lines (UF-52 and UF-53) also exhibited lemon-colored leaves; they were selected to produce additional segregating populations to validate the above inferred mode of inheritance for leaf background color. Progeny of crosses UF-52×UF-317 and UF-53×‘Gingerland’ (Table 2; cross no. 17 and 18) segregated in 1 (lemon): 1 (green) (*P*=0.325–0.455). When UF-52 and ‘Miss Muffet’ were crossed (Table 2; cross no. 19), their progeny segregated in an anticipated ratio of 3 (lemon): 1 (green) (*P*=0.611). These results support the above inference that a single nuclear locus controls leaf background color and also suggest that UF-52 and UF-53 have a heterozygous genotype at the leaf background color locus.

We propose *LEM* as the gene symbol for the dominant allele controlling the lemon background color and *lem* for the recessive allele controlling the green leaf background color. Therefore, ‘Miss Muffet’, UF-52, and UF-53 have a heterozygous genotype of *LEMlem*, while ‘Aaron’, ‘Candidum’, ‘Fairytale Princess’, ‘Gingerland’, ‘Fla. Moonlight’, ‘Red Flash’, and UF-317 possess a recessive genotype of *lemlem.*

### Genetic relationship between leaf background color and leaf shape

Previous reports showed that leaf shapes in caladium are controlled by a single locus with two co-dominant alleles, *F* and *f*, which form three genotypes *FF*, *Ff*, and *ff* conferring fancy, lance, and strap leaves, respectively.^3^ The lance-leaved ‘Gingerland’ has the *Ff* genotype and fancy-leaved ‘Miss Muffet’ and UF-52 has a *FF* genotype.^4,6^

In this study, progeny of the cross between ‘Gingerland’ and ‘Miss Muffet’ (Table 3; cross no. 2 and 3) showed a segregation ratio of 1 (fancy, lemon): 1 (fancy, green): 1 (lance, lemon): 1 (lance, green) (*P*=0.353–0.537), as expected for two independently inherited loci. When UF-52 was crossed with ‘Gingerland’ (Table 3; cross no. 18), the same segregation ratio [1 (fancy, lemon): 1 (fancy, green): 1 (lance, lemon): 1 (lance, green) (*P*=0.360)] was observed. These results indicated that leaf background color and leaf shape were not genetically linked.

### Genetic relationship between leaf background color and spotting

Previous studies showed that leaf spotting in caladium is controlled by a single locus with two alleles, the dominant *s* allele for spotted leaves and the recessive *s* allele for non-spotted leaves.^4^ And, the spotted ‘Miss Muffet’ and ‘Gingerland’ both have a heterozygous genotype *Ss*, and non-spotted ‘Fla. Moonlight’ and ‘Aaron’ possess a homozygous genotype *ss*.^4,6^

In this study, both leaf spotting and leaf background color segregated in six crosses (Table 4). Progeny of crosses between ‘Miss Muffet’ and ‘Gingerland’ (Table 4; cross no. 2 and 3) did not segregate in 3 (spotted, lemon): 3 (spotted, green): 1 (non-spotted, lemon): 1 (non-spotted, green) (*P*<0.001) as expected for two independently inherited loci. Progeny of the crosses between ‘Miss Muffet’ and ‘Candidum’ (Table 4; cross no. 4 and 5) did not segregate in 1 (spotted, lemon): 1 (spotted, green): 1 (non-spotted, lemon): 1 (non-spotted, green) (*P*<0.001). Neither did the progeny of the crosses ‘Fla. Moonlight’×‘Miss Muffet’ and ‘Aaron’×‘Miss Muffet’ (*P*<0.001) (Table 4; cross no. 15 and 16). These results suggested that leaf spotting and leaf background color were genetically linked.

In the four pseudo backcrosses (Table 4; cross no. 4, 5, 15 and 16), two types of progeny (spotted and green, and non-spotted and lemon) were significantly more abundant than expected for independently inherited traits, suggesting that the spotted allele (*S*) was in coupling phase with the green background color allele (*lem*), and the non-spotted allele (*s*) was in coupling phase with the lemon background color (*LEM*).

In cross no. 2 and no. 3 (Table 4), only one type of recombinants (non-spotted and green progeny) could be identified. In the four pseudo BC_1_ populations (Table 4; cross no. 4, 5, 15 and 16), two types of recombinants (spotted and lemon progeny, and non-spotted and green progeny) were present. These recombinant progeny were used to calculate the recombination frequencies between leaf spotting and leaf background color loci, *S* and *LEM*. The recombination frequency between *S* and *LEM* in the six crosses varied from 3.30 to 9.02%, and was averaged to be 5.37% (Table 4).

### Genetic relationship between leaf background color and main vein color

Leaf background color and main vein color segregated in five crosses (Table 5). Caladium main vein colors are controlled by a single nuclear locus with three alleles, *V*^r^ for red veins>*V*^w^ for white veins>*V*^g^ for green veins.^3^ The genotype of ‘Candidum’ and UF-317 for leaf main vein color was determined to be *V*^g^*V*^g^, and the genotype of ‘Miss Muffet’ and UF-52 was inferred to be *V*^w^*V*^g^.^6^

When ‘Miss Muffet’ was crossed with ‘Gingerland’ (Table 5; cross no. 2 and 3), their progeny did not segregate in 3 (white veined, lemon): 3 (white veined, green): 1 (green veined, lemon): 1 (green veined, green), as expected for two loci under independent assortment. Progeny of two pseudo BC_1_ crosses, ‘Miss Muffet’ and ‘Candidum’ (Table 5; cross no. 4 and 5), also did not segregate in 1 (white veined, lemon): 1 (white veined, green): 1 (green veined, lemon): 1 (green veined, green), as expected for independent inheritance of leaf background color and leaf main vein color (*P*<0.001). Neither did the segregation in the cross UF-52×UF-317 (Table 5; cross no. 17) (*P*<0.001). These results indicated a genetic linkage between leaf main vein color and leaf background color.

In three pseudo BC_1_ populations (Table 5; cross no. 4, 5 and 17), significantly more progeny with white vein and lemon leaf background or with green vein and green leaf background were observed than expected, indicating a repulsion phase between white veined allele (*V*^*w*^) and green leaf background allele (*lem*).

In cross no. 2 and 3 (Table 5), only one type of recombinants could be identified and they showed green veins and lemon background color. The recombination frequencies between *V* and *LEM* loci were estimated to be between 6.02 and 10.37%. In the three pseudo BC_1_ populations (Table 5; cross no. 4, 5 and 17), two types of recombinants were present, white-veined progeny with green leaf background color, and green-veined progeny with lemon color. The recombination frequencies between *V* and *LEM* loci in these crosses varied from 3.29 to 5.51%. The average recombination frequency from these crosses was 5.80% (Table 5).

### Genetic relationship between leaf background color and leaf rugosity

Both rugose leaf and leaf background color segregated in the progeny of four crosses (Table 6). A previous study has shown that leaf rugosity in ‘Miss Muffet’ and other caladium cultivars is controlled by a single nuclear locus with a dominant rugose leaf allele (*RLF*) and a recessive non-rugose leaf allele (*rlf*).^6^ The genotype of ‘Miss Muffet’ (rugose) and ‘Candidum’ (non-rugose) for the leaf rugosity locus is heterozygous (*RLFrlf*) and homozygous recessive (*rlfrlf)*, respectively.^6^

Progeny of the crosses between ‘Miss Muffet’ and ‘Gingerland’ (Table 6; cross no. 2 and 3) did not segregate in 3 (rugose, lemon): 3 (rugose, green): 1 (non-rugose, lemon): 1 (non-rugose, green), as expected for two independently inherited loci (*P*<0.001). Similarly, the segregation of rugose leaf and leaf background color in the progeny of pseudo BC_1_ populations of ‘Miss Muffet’ and ‘Candidum’ (Table 6; cross no. 4 and 5) deviated from 1 (rugose, lemon): 1 (rugose, green): 1 (non-rugose, lemon): 1 (non-rugose, green), as expected for two independently inherited loci (*P*<0.001).

In cross no. 2 and 3 (Table 6), one type of recombinants was identified, and they were non-rugose and had the green leaf background color. The recombination frequencies in these crosses were estimated to be 14.47 to 15.79% (Table 6). In two pseudo BC_1_ populations (Table 5; cross no. 4 and 5), two types of recombinants were identified: rugose and lemon progeny, and non-rugose and green progeny. The recombination frequencies in these two BC_1_ crosses were 10.86 to 15.48%. The average recombination frequency between the rugose leaf and the leaf background color loci in these populations was 14.15% (Table 6).

### Genetic distance and gene order among main vein color, leaf spotting, rugose leaf, and leaf background color

**Figure 2:**
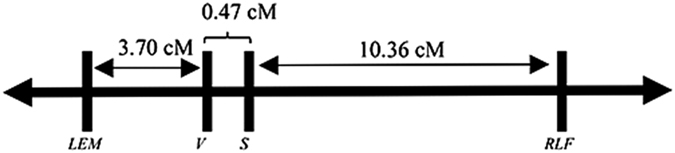
Genetic linkage map of four loci for leaf main vein color (*V*), spotting (*S*), rugosity (*RLF*) and leaf background color (*LEM*). Genetic distances (cM) were calculated from the recombination frequencies among *V*, *S*, *RLF* and *LEM* in two pseudo BC_1_ populations of ‘Miss Muffet’ (*V*^w^ *s rlf LEM*//*V*^*g*^ *S RLF lem*) and ‘Candidum’ (*V*^g^ *s rlf lem*// *V*^g^ *s rlf lem*) using the Kosambi mapping function.

Progeny of the crosses between ‘Miss Muffet’ and ‘Candidum’ segregated for four characters, main vein color, leaf spotting, rugose leaf, and leaf background color (Table 7). These progeny provided an opportunity to determine the genetic relationships among the loci controlling these characters. Supplementary Figure S1 and Table 7 illustrated the potential types of gametes and progeny that could be produced by ‘Miss Muffet’ and ‘Candidum’ when they were crossed. Since ‘Candidum’ is homozygous recessive at the four loci controlling these traits, the number of recombinants observed in the crosses could be used directly to calculate recombination frequencies between the four loci. The observed recombination frequencies between these loci ranged from 0.47% (between *V* and *S*) to 14.84% (between *LEM* and *RLF*; Table 7). And the order of the four loci were determined to be: *LEM–V–S–RLF.* A genetic map of the four loci based on these recombination frequencies and the Kosambi mapping function was shown in Figure 2.

## Discussion

Creating novel leaf characteristics and new combinations of existing leaf characteristics have been a very important objective in caladium breeding. A better understanding of the mode of inheritance for caladium leaf characteristics is of tremendous value to caladium breeding. This study revealed the mode of inheritance for leaf background colors in caladiums, inferred the genotypes of important caladium cultivars and breeding lines at leaf background color locus, and developed a genetic linkage map of four loci controlling leaf main vein color, spotting, rugosity, and background color. The information gained in this study on the inheritance of these traits could empower caladium breeders to better plan parental combinations, breeding populations, and selection for developing new caladium cultivars. Results from this study indicated that the lemon-colored cultivar ‘Miss Muffet’ and breeding lines UF-52 and UF-53 are heterozygous at the *LEM* locus. Thus, it is expected that when one of them is crossed with a caladium cultivar/breeding line with green background color, half of their progeny are expected to possess lemon-colored leaves. When the lemon-colored caladiums are crossed, three-fourths of their progeny are expected to have lemon-colored leaves.

The leaf background color locus *LEM* is tightly linked with the leaf main vein color locus *V*, spotting locus *S* and rugosity locus *RLF*, but is independent from the leaf shape locus *F* (Figure 2). Because of this close linkage, approximate 86.1% (1091/1267) of progeny were of the parental types in pseudo BC_1_ populations between ‘Miss Muffet’ and ‘Candidum’, and there were no double crossover progeny (between *RLF* and *S*, and between *S* and *V*), or triple crossover progeny (between *RLF* and *S*, between *S* and *V*, and between *V* and *LEM*) in the populations. There were very few progeny resulting from the double crossover II (between *V* and *S*, and between *V* and *LEM*) (1/1267), double crossover III (between *RLF* and *S*, and between *LEM* and *V*) (7/1267), single crossover III (between *LEM* and *V*) (40/1267), or single crossover I (*S* and *V*) (5/1267) (Table 7, Supplementary Figure S1). The tight linkage among these four traits and the scarcity of recombinants among them suggest that large breeding populations are required to develop new caladium cultivars with new combinations of these four leaf characteristics.

Leaf background color is an important characteristic in many ornamental aroids. In *Aglaonema*, *Colocasia,* and *Dieffenbachia*, green, lemon, yellow, and black leaves have been observed in many cultivars.^16,17^ However, knowledge on the mode of inheritance for leaf background colors in these aroids has been lacking. Previous inheritance studies have shown that caladium and other aroids seem to share similar modes of inheritance for foliar characters.^3,18,19^ Knowledge gained on the mode of inheritance of leaf background color in caladium may be useful for study of the inheritance of leaf background colors in other aroids.

So far, a number of genes for leaf background colors have been mapped in several plant species.^20^ A single dominant gene *Pr,* encoding a R2R3 MYB transcription factor, controls anthocyanin accumulation patterns and purple leaves in *Brassica*.^21^ The yellow leaf trait in rice (*Oryza sativa)* is controlled by a recessive gene *leaf 8* (*ygl8*), which reduce the content of total chlorophyll in rice leaves.^22^ Guan *et al.* ^23^ showed that the yellow leaf trait in maize (*Zea mays*) is controlled by recessive gene *leaf-1* (*ygl1-1*) that causes an abnormal chloroplast development in maize. These studies will be very useful for genetic mapping, molecular cloning, and functional analysis of the *LEM* gene that controls the leaf background color in caladium.

## Figures and Tables

**Table 1:** Phenotypes and genotypes (inferred) of seven commercial caladium cultivars and three breeding lines used as parents for crosses performed in this study

*Cultivar/breeding line*	*Leaf spotting*		*Leaf shape*		*Main vein color*		*Leaf rugosity*		*Leaf background color*	
	*Phenotype*	*Genotype*^a^	*Phenotype*	*Genotype*^a^	*Phenotype*	*Genotype*^a^	*Phenotype*	*Genotype*^a^	*Phenotype*	*Genotype*^b^
‘Aaron’	No	*ss*	Fancy	*FF*	White	*V*^w^*V*^w^	No	*rlfrlf*	Green	*lemlem*
‘Candidum’	No	*ss*	Fancy	*FF*	Green	*V*^g^*V*^g^	No	*rlfrlf*	Green	*lemlem*
‘Fairytale Princess’	No	*ss*	Lance	*Ff*	Red	*V*^r^*V*^g^	No	*rlfrlf*	Green	*lemlem*
‘Gingerland’	Yes	*Ss*	Lance	*Ff*	White	*V*^w^*V*^g^	Yes	*RLFrlf*	Green	*lemlem*
‘Miss Muffet’	Yes	*Ss*	Fancy	*FF*	White	*V*^w^*V*^g^	Yes	*RLFrlf*	Lemon	*LEMlem*
‘Fla. Moonlight’	No	*ss*	Fancy	*FF*	White	*V*^w^*V*^g^	No	*rlfrlf*	Green	*lemlem*
‘Red Flash’	Yes	*Ss*	Fancy	*FF*	Red	*V*^*r*^*V*^*g*^	No	*rlfrlf*	Green	*lemlem*
UF-52	No	*ss*	Fancy	*FF*	White	*V*^w^*V*^g^	No	*rlfrlf*	Lemon	*LEMlem*
UF-53	No	*ss*	Fancy	*FF*	White	*V*^w^*V*^g^	No	*rlfrlf*	Lemon	*LEMlem*
UF-317	Yes	*Ss*	Fancy	*FF*	Green	*V*^g^*V*^g^	Yes	*RLFrlf*	Green	*lemlem*

a Genotypes of these cultivars or breeding lines for leaf shape, main vein color, leaf spotting, and leaf rugosity were determined previously,^3–6^ and they were reconfirmed in this study.

b Inferred in this study.

**Table 2:** Segregation of leaf background color (lemon or green) in 19 caladium crosses

*Cross [cross no.]*	*Caladium progeny (no.)*		*Expect. ratio*^a^	*Chi-square*	
	*Lemon (LEM_)*^b^	*Green (lemlem)*^b^		χ^*2*^	P*-value*
‘Miss Muffet’ (*LEMlem*)×self (*LEMlem*) [1]	248	76	3:1	0.412	0.521
‘Miss Muffet’ (*LEMlem*)×‘Gingerland’ (*lemlem*) [2]	124	142	1:1	1.242	0.265
‘Gingerland’ (*lemlem*)×‘Miss Muffet’ (*LEMlem*) [3]	165	182	1:1	0.833	0.361
‘Miss Muffet’ (*LEMlem*)×‘Candidum’ (*lemlem*) [4]	299	309	1:1	0.149	0.685
‘Candidum’ (*lemlem*)×‘Miss Muffet’ (*LEMlem*) [5]	324	335	1:1	0.184	0.668
‘Miss Muffet’ (*LEMlem*)×‘Red Flash’ (*lemlem*) [6]	13	16	1:1	0.310	0.577
‘Aaron’ (*lemlem*)×‘Miss Muffet’ (*LEMlem*) [7]	104	85	1:1	1.910	0.167
‘Fla. Moonlight’ (*lemlem*)×‘Miss Muffet’ (*LEMlem*) [8]	52	39	1:1	1.857	0.173
‘Gingerland’ (*lemlem*)×self [9]	0	236	0:1		
‘Candidum’ (*lemlem*)×self [10]	0	108	0:1		
‘Gingerland’ (*lemlem*)×‘Candidum’ (*lemlem*) [11]	0	187	0:1		
‘Candidum’ (*lemlem*)×‘Gingerland’ (*lemlem*) [12]	0	193	0:1		
‘Gingerland’ (*lemlem*)×‘Red Flash’ (*lemlem*) [13]	0	124	0:1		
‘Red Flash’ (*lemlem*)×‘Gingerland’ (*lemlem*) [14]	0	29	0:1		
‘Fla. Moonlight’ (*lemlem*)×‘Fairytale Princess’ (*lemlem*) [15]	0	28	0:1		
‘Aaron’ (*lemlem*)×UF-317 (*lemlem*) [16]	0	213	0:1		
UF-52 (*LEMlem*)×UF-317 (*lemlem*) [17]	68	77	1:1	0.559	0.455
UF-53 (*LEMlem*)×‘Gingerland’ (*lemlem*) [18]	37	29	1:1	0.970	0.325
UF-52 (*LEMlem*)×‘Miss Muffet’ (*LEMlem*) [19]	49	14	3:1	0.259	0.611

a Segregation ratios expected for single dominant nuclear genes controlling traits.

b Two possible genotypes (*LEMLEM* or *LEMlem*, generalized as *LEM_* here) exhibit the lemon background color, and the recessive genotype (*lemlem*) displays the green background color.

**Table 3:** Joint segregation of leaf shape (fancy or lance) and leaf background color (lemon or green) in progeny of three caladium crosses

*Cross [cross no.]*	*Progeny (no.)*				*Expect. ratio*^a^	χ^*2*^ (P)
	*Fancy (FF)*		*Lance (Ff)*			
	*Lemon (LEM_)*^b^	*Green (lemlem)*	*Lemon (LEM_)*^b^	*Green (lemlem)*		
‘Miss Muffet’ (*FF LEMlem*)×‘Gingerland’ (*Ff lemlem*) [2]	64	79	60	63	1:1:1:1	3.263 (0.353)
‘Gingerland’ (*Ff lemlem*)×‘Miss Muffet’ (*FF LEMlem*) [3]	89	87	76	95	1:1:1:1	2.176 (0.537)
UF-53 (*FF LEMlem*)×‘Gingerland’ (*Ff lemlem*) [18]	21	18	16	11	1:1:1:1	3.212 (0.360)

a Segregation ratio expected for independent inheritance between leaf shape and leaf background color.

b Two possible genotypes (*LEMLEM* or *LEMlem*, generalized as *LEM_* here) produce the lemon background color, and only the recessive genotype (*lemlem*) produces the green background color.

**Table 4:** Joint segregation of leaf spotting (present or absent) and leaf background color (lemon or green) in progeny of five caladium crosses

*Cross [cross no.]*	*Caladium progeny (no.)*^a^				*Expect. ratio*^b^	*χ*^*2*^ (P)	*Recombination (%)*
	*Spotted (S_)*		*Non-spotted (ss)*				
	*Lemon (LEM_)*	*Green (lemlem)*	*Lemon (LEM_)*	*Green (lemlem)*			
‘Miss Muffet’ (*S lem*//*s LEM*)×‘Gingerland’ (*S lem*//*s lem*) [2]	70	130	54	12	3:3:1:1	47.618 (*P*<0.001)	9.02^c^
‘Gingerland’ (*S lem*//*s lem*)×‘Miss Muffet’ (*S lem*//*s LEM*) [3]	103	172	62	10	3:3:1:1	65.903 (*P*<0.001)	5.76^c^
‘Miss Muffet’ (*S lem*//*s LEM*)×‘Candidum’ (*s lem*//*s lem*) [4]	6	294	293	15	1:1:1:1	527.171 (*P*<0.001)	3.45^d^
‘Candidum’ (*s lem*//*s lem*)×‘Miss Muffet’ (*S lem//s LEM*) [5]	17	323	307	12	1:1:1:1	548.958 (*P<*0.001)	4.40^d^
‘Fla. Moonlight’ (*s lem//s lem*)×‘Miss Muffet’ (*S lem//s LEM)* [15]	2	38	50	1	1:1:1:1	82.582 (*P<*0.001)	3.30^d^
‘Aaron’ (*s lem//s lem*)×‘Miss Muffet’2 (*S lem//s LEM*) [16]	4	83	96	8	1:1:1:1	153.286 (*P<*0.001)	6.28^d^
						Average	5.37 (±2.15)

a Two possible genotypes (*LEMLEM* or *LEMlem*, generalized as *LEM_* here) produce the lemon background color, and only the recessive genotype (*lemlem*) produces the green background color. Two possible genotypes (*SS* or *Ss*, generalized as *S_* here) produce the spotted progeny, and only the recessive genotype (*ss*) produces non-spotted progeny.

b Segregation ratio expected for independent inheritance between leaf spotting and leaf background color.

c Recombination frequencies in these populations were calculated as follows: [(no. of non-spotted and green progeny/total no. of progeny)×2]×100.

d Recombination frequencies in these pseudo BC_1_ populations were calculated as follows: [(no. of spotted and lemon progeny+no. of non-spotted and green progeny)/total no. of progeny]×100.

**Table 5:** Joint segregation of leaf background color (lemon or green) and main vein color (white or green) in progeny of four caladium crosses

*Cross [cross no.]*	*Caladium progeny (no.)*				*Expect. ratio*^a^	*χ*^*2*^ *(P)*	*Recombination (%)*
	*White veined (V*^*w*^*_)*^b^		*Green veined (V*^*g*^*V*^*g*^)				
	*Lemon (LEM_)*^b^	*Green (lemlem)*	*Lemon (LEM_)*^b^	*Green (lemlem)*			
‘Miss Muffet’ (*V*^w^ *LEM//V*^g^ *lem*)×‘Gingerland’ (*V*^w^ *lem//V*^g^ *lem*) [2]	116	78	8	64	3:3:1:1	55.003 (*P* <0.001)	6.02^c^
‘Gingerland’ (*V*^*w*^ *lem//V*^*g*^ *lem*)×‘Miss Muffet’ (*V*^*w*^ *LEM//V*^*g*^ *lem*) [3]	147	99	18	83	3:3:1:1	83.906 (*P* <0.001)	10.37^c^
‘Miss Muffet’ (*V*^*w*^ *LEM//V*^*g*^ *lem*)×‘Candidum’ (*V*^*g*^ *lem//V*^*g*^ *lem*) [4]	293	14	6	295	1:1:1:1	530.855 (*P* <0.001)	3.29^d^
‘Candidum’ (*V*^g^ *lem//V*^g^ *lem*)×‘Miss Muffet’ (*V*^w^ *LEM//V*^g^ *lem*) [5]	310	11	14	324	1:1:1:1	563.416 (*P* <0.001)	3.79^d^
UF-52 (*V*^w^ *LEM//V*^g^ *lem*)×UF-317 (*V*^g^ *lem//V*^g^ *lem*) [17]	64	5	3	73	1:1:1:1	115.685 (*P* <0.001)	5.51^d^
						Average	5.80 (±2.80)

a Two possible genotypes (*V*^w^*V*^w^ and *V*^w^*V*^g^, generalized as *V*^w^*_* here) in these crosses produce white-veined progeny, and the recessive genotype (*V*^g^*V*^g^) produces green-veined progeny. Two possible genotypes (*LEMLEM* or *LEMlem*, generalized as *LEM_* here) produce the lemon background color, and the recessive genotype (*lemlem*) produces the green background color.

b Segregation ratio expected for independent inheritance between leaf main vein color and leaf background color.

c Recombination frequencies in these populations were calculated as follows: [(no. of green-veined and lemon progeny/total no. of progeny)×2]×100.

d Recombination frequencies in these pseudo BC_1_ populations were calculated as follows: [(no. of white-veined and green progeny+no. of green-veined and lemon progeny)/total no. of progeny]×100.

**Table 6:** Joint segregation of leaf background color (lemon or green) and leaf rugosity (rugose or flat) in progeny of four caladium crosses

*Cross [cross no.]*	*Caladium progeny (no.)*				*Expect. Ratio*^a^	*χ*^*2*^ (P)	*Recombination (%)*
	*Rugose (RLF_)*^b^		*Non-rugose (rlfrlf)*				
	*Lemon (LEM_)*^b^	*Green (lemlem)*	*Lemon (LEM_)*^b^	*Green (lemlem)*			
‘Miss Muffet’ (*LEM rlf//lem RLF*)×‘Gingerland’ (*lem RLF//lem rlf*) [2]	41	94	40	15	3:3:1:1	69.832 (*P*<0.001)	15.79^c^
‘Gingerland’ (*lem RLF//lem rlf*)×‘Miss Muffet’ (*LEM rlf//lem RLF*) [3]	69	106	43	17	3:3:1:1	73.681 (*P*<0.001)	14.47^c^
‘Miss Muffet’ (*LEM rlf//lem RLF*)×‘Candidum’ (*lem rlf //lem rlf*) [4]	29	272	270	37	1:1:1:1	372.882 (*P*<0.001)	10.86^d^
‘Candidum’ (*lem rlf //lem rlf*)×‘Miss Muffet’ (*LEM rlf//lem RLF*) [5]	62	295	262	40	1:1:1:1	318.924 (*P*<0.001)	15.48^d^
						Average	14.15 (±2.26)

a Segregation ratio expected for independent inheritance between leaf rugosity and leaf background color.

b Two possible genotypes (*RLFRLF* or *RLFrlf*, generalized as *RLF_* here) produce rugose progeny, and the recessive genotype (*rlfrlf*) produces the non-rugose progeny. Two possible genotypes (*LEMLEM* or *LEMlem*, generalized as *LEM_* here) produce the lemon background color, and the recessive genotype (*lemlem*) produces green background color.

c Recombination frequencies in these populations were calculated as follows: [(no. of non-rugose and green progeny/total no. of progeny)×2]×100.

d Recombination frequencies in these pseudo BC_1_ populations were calculated as follows: [(no. of white-veined and green progeny+no. of green-veined and lemon progeny)/total no. of progeny]×100.

**Table 7:** Joint segregation of four foliar traits, main vein color, spotting, rugosity and background color in progeny of ‘Miss Muffet’ (*V*^w^ *s LEM rlf//V*^g^ *S lem RLF*) and ‘Candidum’ (*V*^*g*^ *s lem rlf //Vg s lem rlf*)

*Type of progeny*	*Caladium progeny*				*No. of progeny*
	*Main vein color*	*Leaf spotting*	*Leaf background color*	*Leaf rugosity*	
Parental types	Green (*V*^g^*V*^g^)	Spotted (*Ss*)	Green (*lemlem*)	Rugose (*RLFrlf*)	563
	White (*V*^w^*V*^g^)	Non-spotted (*ss*)	Lemon (*LEMlem*)	Non-rugose (*rlfrlf*)	528
Single crossover I (between *RLF* and *S*)	White (*V*^w^*V*^g^)	Non-spotted (*ss*)	Lemon (*LEMlem*)	Rugose (*RLFrlf*)	71
	Green (*V*^g^*V*^g^)	Spotted (*Ss*)	Green (*lemlem*)	Non-rugose (*rlfrlf*)	56
Single crossover II (between *S* and *V*)	White (*V*^w^*V*^g^)	Spotted (*Ss*)	Lemon (*LEMlem*)	Rugose (*RLFrlf*)	4
	Green (*V*^g^*V*^g^)	Non-spotted (*ss*)	Green (*lemlem*)	Non-rugose (*rlfrlf*)	1
Single crossover III (between *LEM* and *V*)	Green (*V*^g^*V*^g^)	Spotted (*Ss*)	Lemon (*LEMlem*)	Rugose (*RLFrlf*)	19
	White (*V*^w^*V*^g^)	Non-spotted (*ss*)	Green (*lemlem*)	Non-rugose (*rlfrlf*)	21
Double crossover I (between *RLF* and *S*, and between *S* and *V*)	White (*V*^w^*V*^g^)	Spotted (*Ss*)	Lemon (*LEMlem*)	Non-rugose (*rlfrlf*)	0
	Green (*V*^g^*V*^g^)	Non-spotted (*ss*)	Green (*lemlem*)	Rugose (*RLFrlf*)	0
Double crossover II (between *S* and *V*, and between *V* and *LEM*)	Green (*V*^g^*V*^g^)	Non-spotted (*ss*)	Lemon (*LEMlem*)	Non-rugose (*rlfrlf*)	1
	White (*V*^w^*V*^g^)	Spotted (*Ss*)	Green (*lemlem*)	Rugose (*RLFrlf*)	0
Double crossover III (between *RLF* and *S,* and between *LEM* and *V*)	Green (*V*^g^*V*^g^)	Spotted (*Ss*)	Lemon (*LEMlem*)	Non-rugose (*rlfrlf*)	3
	White (*V*^w^*V*^g^)	Non-spotted (*ss*)	Green (*lemlem*)	Rugose (*RLFrlf*)	4
Triple crossover (between *RLF* and S, between *S* and *V*, and between *V* and *LEM*)	Green (*V*^g^*V*^g^)	Non-spotted (*ss*)	Lemon (*LEMlem*)	Rugose (*RLFrlf*)	0
	White (*V*^w^*V*^g^)	Spotted (*Ss*)	Green (*lemlem*)	Non-rugose (*rlfrlf*)	0
					
Total					1267
Recombination frequency between *RLF* and *S*:	10.58%^a^				
Recombination frequency between *S* and *V*:	0.47%^b^				
Recombination frequency between *V* and *LEM*:	3.79%^c^				
Recombination frequency between *RLF* and *LEM*:	14.84%^d^				

a Recombination frequency between *RLF* (leaf rugosity) and *S* (leaf spotting) was calculated using the following formula: (no. of progeny from single crossover I + no. of progeny from double crossover I + no. of progeny from double crossover III + no. of progeny from triple crossover)/total number of progeny×100.

b Recombination frequency between *S* (leaf spotting) and *V* (leaf main vein color) was calculated using the following formula: (no. of progeny from single crossover II + no. of progeny from double crossover I + no. of progeny from double crossover II + no. of progeny from triple crossover)/total number of progeny×100.

c Recombination frequency between *V* and *LEM* (leaf background color) was calculated using the following formula: (no. of progeny from single crossover III + no. of progeny from double crossover II + no. of progeny from double crossover III + no. of progeny from triple crossover)/total number of progeny×100.

d Recombination frequency between *RLF* and *LEM* was calculated using the following formula: (recombination frequency between *RLF* and *S* + recombination frequency between *S* and *V* + recombination frequency between *V* and *LEM*).
