# Supplementary figures and images for: A single gene controls leaf background color in caladium (Araceae) and is tightly linked to genes for leaf main vein color, spotting and rugosity
Source: Hortic Res. 2017 Jan 4;4:16067–. doi: 10.1038/hortres.2016.67 (PMC5209669; doi:10.1038/hortres.2016.67)

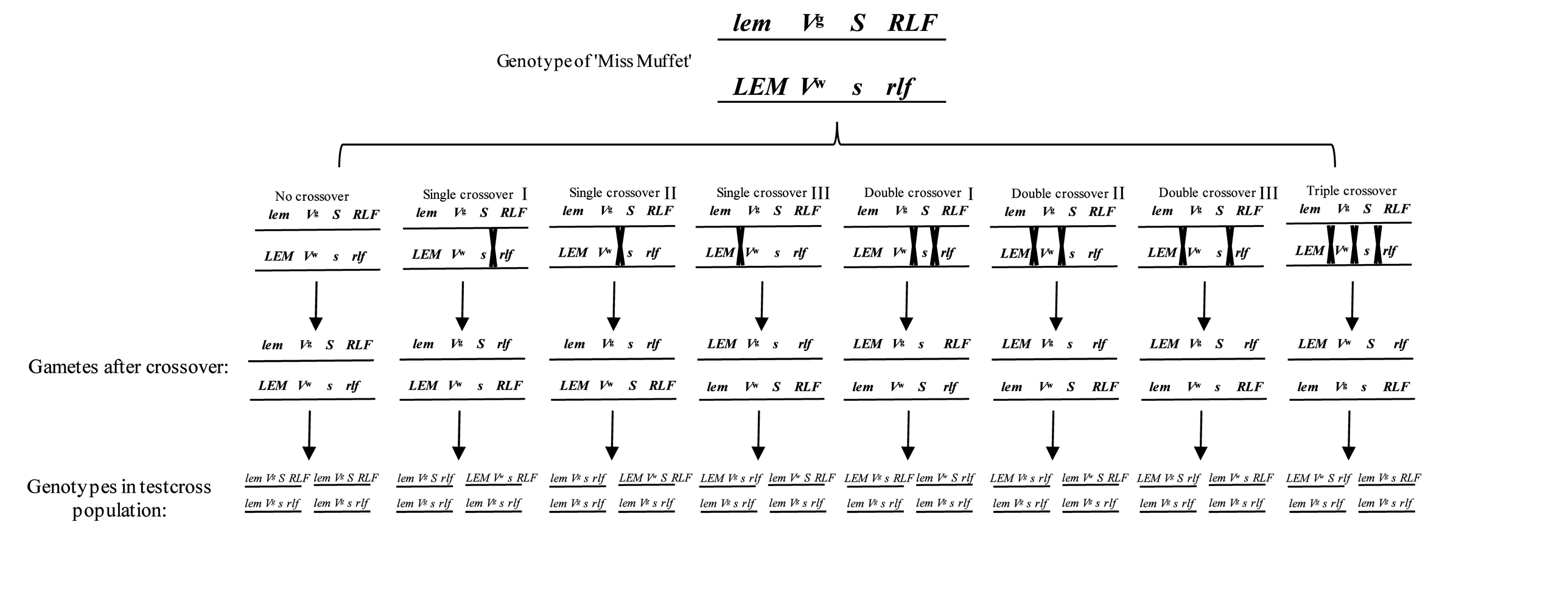

Supplement: Supplementary Figure S1 [file hortres201667-s1.jpg]
